# Identification of Patients with Similar Gait Compensating Strategies Due to Unilateral Hip Osteoarthritis and the Effect of Total Hip Replacement: A Secondary Analysis

**DOI:** 10.3390/jcm10102167

**Published:** 2021-05-17

**Authors:** Stefan van Drongelen, Bernd J. Stetter, Harald Böhm, Felix Stief, Thorsten Stein, Andrea Meurer

**Affiliations:** 1 Dr. Rolf M. Schwiete Research Unit for Osteoarthritis, Department of Orthopedics (Friedrichsheim), University Hospital Frankfurt, Goethe University, 60528 Frankfurt/Main, Germany; 2 Institute of Sports and Sports Science, Karlsruhe Institute of Technology, 76131 Karlsruhe, Germany; bernd.stetter@kit.edu (B.J.S.); thorsten.stein@kit.edu (T.S.); 3 Orthopedic Hospital for Children, Behandlungszentrum Aschau gGmbH, 83229 Aschaui im Chiemgau, Germany; h.boehm@bz-aschau.de; 4 Department of Orthopedics (Friedrichsheim), University Hospital Frankfurt, Goethe University, 60528 Frankfurt/Main, Germany; felix.stief@kgu.de (F.S.); andrea.meurer@kgu.de (A.M.)

**Keywords:** unilateral hip osteoarthritis, classification, 3D gait analysis, total hip replacement, cluster analysis

## Abstract

Despite good clinical functional outcome, deficits in gait biomechanics exist 2 years after total hip replacement surgery. The aims of this research were (1) to group patients showing similar gait adaptations to hip osteoarthritis and (2) to investigate the effect of the surgical treatment on gait kinematics and external joint moments. In a secondary analysis, gait data of 51 patients with unilateral hip osteoarthritis were analyzed. A k-means cluster analysis was performed on scores derived via a principal component analysis of the gait kinematics. Preoperative and postoperative datasets were statistically tested between clusters and 46 healthy controls. The first three principal components incorporated hip flexion/extension, pelvic tilt, foot progression angle and thorax tilt. Two clusters were discriminated best by the peak hip extension during terminal stance. Both clusters deviated from healthy controls in spatio-temporal, kinematic and kinetic parameters. The cluster with less hip extension deviated significantly more. The clusters improved postoperatively but differences to healthy controls were still present one year after surgery. A poor preoperative gait pattern in patients with unilateral hip osteoarthritis is associated with worse gait kinematics after total hip replacement. Further research should focus on the identification of patients who can benefit from an adapted or individualized rehabilitation program.

## 1. Introduction

Hip osteoarthritis (OA) is one of the most common degenerative diseases of the musculoskeletal system [1] and a leading cause for disability in the older population. The prevalence of hip OA depends on age and sex: women are more often affected than men [2,3]. Hip OA is associated with joint pain and functional limitations [4]. When conservative therapy is not helping anymore and the personal suffering is too much, a total hip replacement (THR) is performed to reduce the pain and restore normal activity. Despite good clinical functional outcome [5] and a recovery of spatio-temporal gait parameters [6], gait kinematics and gait kinetics are not restored completely even two years after surgery [7,8].

Andriacchi and Mündermann [9] demonstrated that the external knee adduction moment (KAM) during walking is a predictor of the progression of cartilage degeneration in the medial joint compartment and the development of OA in the knee joint. Patients with hip and knee pathology present alterations in gait which have an effect on joint moments and loading [10,11]. Unfavorable kinematics and kinetics lead to higher joint loads in neighboring joints and are believed to play an important role in the development of OA in the neighboring joints in hip OA patients [12,13].

Preoperative identification of persons at risk for developing OA in neighboring joints is necessary to optimize rehabilitation for patients undergoing THR and to reduce the costs to the health care system for treating OA. Determinants that can provide an estimate of the surgical success have been subject to research since the late nineties [14]. Most research studied determinants of postoperative pain and physical function measured by questionnaires. Since self-reported questionnaires are not always a good representation of the actual functional status, researchers tried to predict the ambulatory status by the Timed Up and Go Test or the 6-Minute Walk Test [15,16]. However, ambulatory status gives no information on the quality of walking that can be measured by the gait kinematics and gait kinetics [17,18]. Foucher and Freels [19] linked the preoperative abductor strength to higher adduction and external rotation moments during postoperative gait.

Previous studies often only considered the mean values of a patient group for their evaluation. This usually hides specific clinical findings of the pathology and individual compensating strategies during gait, as expressions in different directions are averaged out. In the last couple of years modern data science methods have become increasingly important to support and standardize researchers’ and clinicians’ decisions when assessing movement abnormalities and/or identifying changes due to orthopedic or physiotherapeutic interventions [20,21]. These methods can process large quantities of data without entirely depending on a priori knowledge of predictive factors like age and gender for the detection of distinct movement patterns [20]. Patients having similar pathologies can be grouped based on regularities in the data, using so-called unsupervised machine learning methods, such as the popular k-means clustering technique [21,22]. Cluster analysis has been used to identify functional groups in patients walking with crouch gait [23], patients with flexible flatfeet [24], swimming [25] and running [26,27].

The first aim of this study was to group unilateral hip OA patients based on 3D kinematics during preoperative walking. The second aim was to investigate the effect of the surgical treatment on gait kinematics and joint loadings of the previously identified subpopulations in comparison to healthy controls. It was hypothesized that the extent of the preoperative gait pathology has an impact on the postoperative outcome. In other words, patients with more severe gait deviations preoperatively still differ more from healthy controls postoperatively.

## 2. Materials and Methods

In this secondary analysis, data of patients who participated in previous prospective studies in our clinic were analyzed [7,28,29,30]. Furthermore, data from all healthy controls with a similar age distribution who were present in our database were used for comparison.

### 2.1. Participants

Patients who were scheduled for and who received a THR because of pain resulting in functional limitations due to unilateral hip OA were considered for inclusion. The main exclusion criteria of the performed prospective studies were the inability to walk without walking aids, inflammatory arthritis, OA of the lower limb joints (other than the affected hip), chronic or neuromuscular diseases, musculoskeletal conditions involving any other lower extremity joints or low back, orthopedic surgeries within the past 6 months and previous joint replacement in the lower extremities. Only data of patients who had a gait analysis in the week before and at least one year after surgery were included. All surgeries were performed, using a lateral approach, the modified minimal invasive Hardinge [31], by experienced orthopedic surgeons. Patients with a body mass index (BMI) >32 kgm^−2^ and patients who walked preoperatively with a speed <0.7 ms^−1^ were excluded from the analyses. In the end, data of 51 symptomatic unilateral hip OA patients were included in the study (Table 1).

Preoperatively, 38 patients scored on average 47.8 (14.4) on the pain section of the Hip disability and Osteoarthritis Outcome Score (HOOS) [32,33], whereas 13 patients from another study scored 55.4 (8.6) on the Harris Hip Score (HHS) [34]. Postoperatively the values for HOOS and HHS were 90.2 (12.0) and 92.6 (10.1), respectively.

Forty-six persons with a similar age distribution were included as a healthy control collective for comparison (Table 1). The control subjects were included if they had no history of orthopedic surgeries or chronic and neuromuscular diseases.

All patients and healthy controls gave written informed consent prior to participation in the original studies.

### 2.2. Gait Analysis

The complete protocol for these studies has been described previously [7,30]. In short, a three-dimensional gait analysis was performed in the week before surgery and one year [30] or two years [7] after surgery. All subjects had to walk barefoot at self-selected speed in the gait laboratory. Kinematic data were collected at 200 Hz (8 MX T10 cameras, VICON Motion Systems, Oxford, UK) and additionally, two AMTI force plates (Advanced Mechanical Technology, Inc., Watertown, MA, USA) were used to synchronously collect ground reaction forces at 1000 Hz.

The marker protocol used for this study was a modified version of the Plug-in-Gait model [35] with additional markers placed on the medial malleolus, medial femoral condyle and trochanter major. This model (called MA) significantly reduced the knee axis cross-talk phenomenon (that is, one joint rotation (e.g., flexion) being interpreted as another (e.g., adduction or varus) due to axis misalignment) suggesting improved accuracy in determining kinematic and kinetic gait parameters compared to the conventional Plug-in-Gait model [35]. Marker trajectories were reconstructed and smoothed with a Woltring filter (MSE 10) using the Vicon Nexus software (version 2.5, VICON Motion Systems, Oxford, UK). In five trials with a clear foot-forceplate-contact gait cycles were identified and kinematic and kinetic variables were obtained. All external joint moments were normalized to body mass and expressed in Nmkg^−1^. Kinematic and kinetic patterns, normalized over the gait cycle, were calculated using Matlab (version R2018b, The Mathworks Inc., Ismaning, Germany).

Based on the clinical relevance the following kinematic parameters were included for further analysis: hip flexion, extension and range of motion (RoM), knee flexion, extension and RoM, pelvic tilt and obliquity, the foot progression angle (FPA—the angle of the long axis of the foot segment relative to the direction of walking), thorax tilt and lean (the lateral displacement of the trunk relative to the supporting limb). For the used system and marker protocol kinematic measurement errors of less than 4° and 6° have been reported for the inter-trial and inter-session variability, respectively [35]. From the external joint moments the hip and knee adduction moment in the frontal plane were selected for further analysis. For these parameters measurement errors of less than 0.06 Nmkg^-1^ have been reported for both the inter-trial and inter-session variability [35].

### 2.3. Principal Component Analysis and Cluster Analysis

In this study a principal component analysis (PCA) was used to identify the most discriminative gait kinematics analog to the method used by Rozumalski et al., [23] and Böhm et al., [24]. The PCA method reduced the dimensionality while preserving most of the variation [21]. Thorax tilt, thorax lean, pelvic tilt, pelvic obliquity, hip flexion-extension, hip adduction-abduction, knee flexion-extension and FPA were selected as initial waveforms for the PCA. These kinematic parameters previously have been reported as typical gait alterations in patients with unilateral hip OA [28,36]. The PCA results in individual scores for each patient that describe how well its waveforms represent the gait patterns.

Based on the scores of the first three eigenvectors of the patients’ gait kinematics, the suitable number of clusters was determined by a hierarchical ward clustering method [37], evaluating the inverse Scree test and the Silhouette coefficients. The k-means clustering technique with the defined number of clusters was applied to identify the patients in the different subgroups. The hierarchical Ward method and non-hierarchical k-means clustering method are established methods for grouping biomechanical data [21] and have proven to be particularly robust [38].

In general, when executing the clustering several times with the same input data, the results can slightly differ. To determine a robust clustering result for our dataset, the clustering algorithm was executed five times.

### 2.4. Statistical Analysis

For the analysis, the following spatio-temporal parameters were calculated as the average value over the included trials: walking speed, step length, cadence, and step width. For all kinematic parameters, except FPA of which the mean over the stance phase was determined, the peak values during the stance phase of gait were calculated. For the kinetic parameters the peak values during the first and second half of the stance phase of gait were calculated.

Shapiro–Wilk tests and visual inspection of the Q-Q plots were used to test for the normal distribution of the anthropometrics and the kinematic and kinetic gait parameters. Unpaired Student’s *t*-tests were used to determine statistical differences between the anthropometrics of controls and patients. A chi-squared test was used to compare gender distribution in the two groups.

After identification of the patients in the different clusters, the selected gait kinematics and kinetics as well as the spatio-temporal parameters were compared using a general linear model (GLM: between variable: clusters, within variable: time (preoperative/postoperative). The parameters of both clusters were statistically compared to the healthy controls using a univariate analysis of variance with walking speed as a covariate for both preoperative and postoperative datasets. As there was a significant difference in height between patients and controls, walking speed was corrected for leg length in this analysis [39].

## 3. Results

### 3.1. Participants

Characteristics of patients and healthy controls are shown in Table 1. Patients were measured preoperatively and again 14.4 (4.6) months after surgery. Patients and healthy controls differed significantly in age, height, weight and BMI.

### 3.2. Characterization of the Identified Clusters

Three patients could not be clearly assigned to one of the clusters, the cluster assignment changed if the clustering was run through five times, and were left out of the analysis. The clustering algorithm was then executed five more times and no changes in the members of each group were observed.

The first three eigenvectors incorporated four kinematic patterns. The first eigenvector that showed the most variance in the gait kinematics consisted of the hip flexion-extension angle and the pelvic tilt. The second eigenvector contained the FPA and the third eigenvector the thorax tilt. The cumulative variance of those first three principal components was 70%.

Two clusters showed the greatest reduction in the inverse scree test as well as the maximum value in the mean silhouette coefficient. The anthropometrics between cluster 1 (*n* = 20) and cluster 2 (*n* = 28) were not significantly different for age and height, but significantly different for weight (74.9 (12.0) kg for cluster 1 vs. 84.0 (10.3) kg for cluster 2, *p* = 0.007) and BMI (25.6 (3.1) kgm^−2^ for cluster 1 vs. 27.5 (2.6) kgm^−2^ for cluster 2, *p* = 0.027). There were more men in cluster 2 but the distribution of the sexes over the clusters was not significantly different (9 men/11 women vs. 19 men/9 women, *p* = 0.113). In addition, the follow-up time did not differ between the clusters (13.2 (3.1) months for cluster 1 vs. 14.4 (4.6) months for cluster 2), whereas the Kellgren–Lawrence score did differ significantly between the clusters (*p* = 0.017).

### 3.3. Gait Parameters of the Identified Clusters and the Effect of Surgery

**Figure 1:**
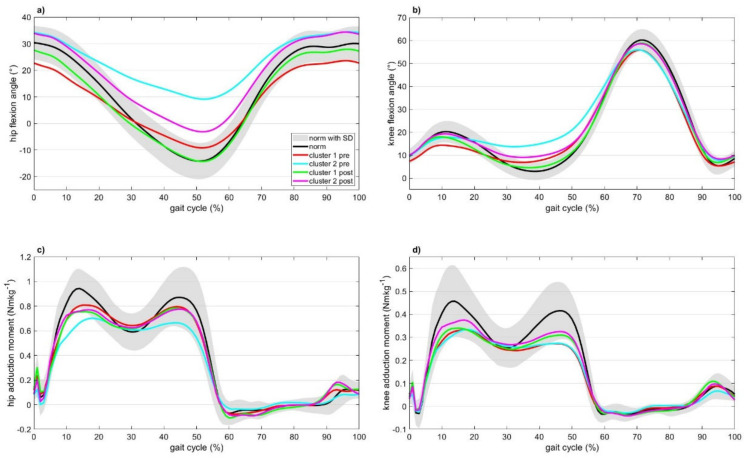
For both clusters and in relation to the healthy controls the preoperative and postoperative (**a**) hip flexion/extension, (**b**) knee flexion/extension, (**c**) external hip adduction moment and (**d**) external knee adduction moment are displayed. Data ar.

The spatio-temporal gait parameters all showed a significant time effect (*p* < 0.001) with step width also showing a significant group effect (Supplementary Table S1). Except for FPA and thorax lean, all kinematic variables showed a significant time effect (a development in the direction of a normal gait pattern). Hip RoM, knee extension and knee RoM showed a group effect (Figure 1). The hip and knee RoM (due to more knee extension) were larger for cluster 1 compared to cluster 2. Hip extension and hip flexion, which are coupled to pelvic tilt, showed a group and time effect with a significant interaction. Regarding the peak joint moments, only a time effect was found for KAM_2: the peak knee adduction moment in the second half of the stance was higher postoperatively in both clusters. An interaction between group and time was found for the hip adduction moment (HAM) during the first half of stance (HAM_1: the peak hip adduction moment during the first half of stance decreased in cluster 1 and increased in cluster 2). The GLM showed significant changes over time for almost all parameters; however, the changes were not of the same magnitude for both clusters.

Preoperative speed and age had an effect on step length and cadence whereas BMI had an effect on walking speed and step width. The existing differences compared to the healthy controls were not present after correcting for speed, age and BMI (Supplementary Table S2). Speed had an effect on hip flexion, hip RoM, knee flexion and knee RoM. Except for knee flexion, pelvic obliquity and FPA, cluster 2 showed significant differences to the healthy controls, whereas cluster 1 only showed differences in hip flexion, hip RoM, knee RoM, pelvic obliquity, thorax tilt and thorax lean. With regard to the joint moments, speed only had an effect on KAM_1 and HAM_1 but only removed the differences between the clusters and healthy controls for KAM_1. BMI had an effect on HAM_1 and HAM_2. After the corrections cluster 1 only had a significant lower KAM_2 whereas cluster 2 showed lower values for KAM_2, and the peak hip adduction moments (HAM_1 and HAM_2) compared to the healthy controls. Overall, cluster 2 showed a gait pattern that deviated more from the healthy controls.

Also postoperatively, speed and age had an effect on step length and cadence. BMI had an effect on step width and age additionally had an effect on walking speed. After the correction, no differences between the clusters and the healthy controls were present (Supplementary Table S2). Speed had a significant effect on hip flexion, hip RoM, knee flexion, knee RoM and pelvic obliquity, whereas BMI had an effect on hip extension, hip flexion and pelvic tilt. Hip extension, hip RoM, knee extension, knee RoM and pelvic tilt were significantly different for cluster 2 compared to the healthy controls but not for cluster 1 compared to the healthy controls. By contrast, only cluster 1 showed significantly more thorax lean compared to the healthy controls. Both clusters still walked with more thorax tilt compared to healthy controls. With regard to the joint moments, speed had a significant effect on KAM_1 and HAM_1. Only cluster 1 showed a significantly lower KAM_2 and HAM_1 compared to the healthy controls. After THR, cluster 2 showed a gait pattern that deviated more from the healthy controls regarding the gait kinematics but not regarding the external joint moments.

## 4. Discussion

The aim of this study was to (1) group unilateral hip OA patients based on the kinematics of their preoperative walking and (2) investigate if differences between the defined clusters were still present one year after surgery. The most relevant finding of the present study are that the two clusters were discriminated best by the peak hip extension during terminal stance and that a poor preoperative gait pattern is associated with worse gait kinematics after THR.

The cluster analysis divided the patients with unilateral hip OA in two clusters that were mainly characterized by the hip flexion-extension angle and the pelvic tilt. Hip flexion-extension and pelvic tilt are coupled movements: with increased pelvic tilt, patients will show more hip flexion and less hip extension (Supplementary Table S1). However, the clusters showed also a functional difference in the hip RoM during gait (cluster 2 had a smaller hip RoM both pre and postoperatively). The two clusters also deviated from each other in the knee kinematics: cluster 2 showed slightly more knee flexion but much less knee extension, which resulted in a lower knee RoM. In hip OA patients (and knee OA patients), reduced flexion of the knee and reduced extension of the hip were found to be closely associated with disability [40]. A closer look at the cluster characteristics showed cluster 2 to have a significantly higher Kellgren–Lawrence score compared to cluster 1. BMI has already been found to be associated with lower knee and hip RoM in patients with early symptomatic OA [41]. In the present study, the cluster with a significantly higher BMI (cluster 2) had a significantly lower hip RoM. Fat stored around the hips and thighs can limit the available hip RoM, however also pain can influence RoM. Doherty et al. [42] reported that pain is caused by stretching and compression of joint structures leading to activation of pain receptors.

Some differences between the clusters and the healthy controls could be attributed to the differences in walking speed: 1.10 (cluster 1) and 1.03 (cluster 2) ms^−1^ preoperatively and 1.21 (cluster 1) and 1.17 (cluster 2) ms^−1^ postoperatively for the clusters compared to 1.30 ms^−1^ for the healthy controls. Ismailidis et al. [43] found that patients with knee OA showed lower knee flexion and lower hip extension compared to healthy controls who walked 0.4 m/s faster than the patients. Although speed had a significant effect on the hip and knee movement in the sagittal plane and the patients walked faster postoperatively, most differences between cluster 2 and the healthy controls were still present after correcting for speed. Hip RoM and hip extension are of clinical importance as a deficit in the hip extension can lead to a persistent pathological gait pattern [44]. These results support our hypothesis that a poor preoperative gait pattern (cluster 2) in patients with unilateral hip OA is associated with worse functional outcome after THR.

At first sight, no differences existed between the clusters regarding the joint moments. However, a closer look on the change over time showed that cluster 2 normalizes KAM_1, HAM_1 and HAM_2 more compared to cluster 1 (values were closer to the healthy controls). Although postoperatively the differences in KAM_1 were due to the difference in speed, KAM_2 and HAM_1 showed a significant difference to the healthy controls for cluster 1 only. These results do not support our hypotheses. Therefore, it might be too easy to say that patients who have a small hip RoM and an insufficient knee extension preoperatively (cluster 2) will have an increased risk for pathological joint moments one year after surgery. By contrast, those patients showed joint moments closer to healthy controls, despite a significant difference in walking speed. Admittedly, cluster 2 showed more variation in the joint loads (larger standard deviation) which indicates that not all patients recover to the same extent.

A limitation of the present study is that with our repeated measures design, the phenomenon regression to the mean (RTM) cannot be ruled out. Although the mean over five valid gait trials was used in the analysis, random error could have been interpreted as real change. However, this does only apply to parameters used for cluster building. Therefore, the conclusion on differences between the clusters with respect to their gait pattern based on parameters not used for cluster building is still supported by the analyses. Besides the applied methods used in the present study, alternative methods can also be used to achieve similar results. The present method has shown its value but it is possible that in combination with other modern data science methods an added value could be achieved when complex boundary conditions like the reconstruction of the femoral offset and leg length after THR surgery are included [21]. Furthermore, our patients were operated on through a lateral approach, which is less muscle sparing compared to direct anterior or posterolateral approaches, which might limit the generalization of the improvement of the gait pattern over time. The effect of a THR approach on gait is still a controversial subject, with conflicting results and differences are likely dependent on recovery time [18,45,46].

## 5. Conclusions

The presented approach can be used to identify and differentiate gait compensation strategies used by unilateral hip OA patients. Hip flexion-extension and pelvic tilt were highlighted as the most important variables in the 3D gait analysis to discriminate these patients. The two clusters showed different changes in their gait pattern. At least one year after THR the gait pattern was not normalized completely as both clusters still showed deviations to the healthy gait pattern. A poor preoperative gait pattern in patients with unilateral hip OA is associated with worse gait kinematics after THR. Further research should focus on a more detailed identification of patients who improve their gait pattern after THR surgery and which patients can benefit from an adapted or individualized rehabilitation program.

## Figures and Tables

**Table 1:** Anthropometric data of the preoperative patients and healthy controls.

Parameter	Patients	Healthy Controls	*p*-Value
Age (years)	60.6 (9.9)	64.2 (7.0)	0.041
Height (m)	1.73 (0.07)	1.69 (0.10)	0.009
Body mass (kg)	80.3 (11.5)	69.0 (12.6)	<0.001
Body Mass Index (kgm^−2^)	26.7 (2.9)	24.2 (2.8)	<0.001
Gender (men/women)	30/21	21/25	0.195

## Data Availability

The data presented in this study are available on request from the corresponding author.

## Supplementary Materials

The following are available online at https://www.mdpi.com/article/10.3390/jcm10102167/s1, Table S1: Preoperative and postoperative spatio-temporal, kinematic and kinetic outcome parameters and the p-values for the general linear model (GLM) of the clusters and Table S2: Preoperative and postoperative spatio-temporal, kinematic and kinetic outcome parameters and the p-values for the univariate analysis of variance (ANOVA) with normalized speed, age and BMI as covariates for comparison of the clusters to the healthy controls.
