# Supplementary material for: Identification of Patients with Similar Gait Compensating Strategies Due to Unilateral Hip Osteoarthritis and the Effect of Total Hip Replacement: A Secondary Analysis
Source: J Clin Med. 2021 May 17;10(10):2167. doi: 10.3390/jcm10102167 (PMC8156609; doi:10.3390/jcm10102167)
Supplement: Supplementary file 1 [file jcm-10-02167-s001.zip › jcm-1188788-supplementary.pdf]

Table S1: Preoperative and postoperative spatio-temporal, kinematic and kinetic outcome parameters and the p-values for the general linear model (GLM) of the clusters.

					GLM		
**Parameter**	**Pre**		**Post**		**Group**	**Time**	**Int.**
	**C1**	**C2**	**C1**	**C2**			
**Spatiotemporal**							
Walking speed (ms^{-1})	1.10 (0.15)	1.03 (0.19)	1.21 (0.11)	1.17 (0.17)	0.214	**0.000**	0.468
Step length (cm)	62.0 (4.3)	58.8 (8.5)	65.5 (5.2)	63.6 (7.4)	0.173	**0.000**	0.385
Cadence (steps per min)	108.1 (9.6)	106.3 (11.0)	111.6 (8.1)	110.6 (9.0)	0.595	**0.000**	0.691
Step width (cm)	9.1 (2.6)	10.7 (3.1)	8.0 (2.6)	9.4 (2.9)	**0.047**	**0.002**	0.746
**Kinematics (°)**							
Hip extension	-9.7 (8.9)	8.7 (6.6)	-14.5 (6.3)	-3.5 (7.9)	**0.000**	**0.000**	**0.001**
Hip flexion	22.6 (5.0)	34.3 (4.6)	27.6 (7.2)	34.0 (4.9)	**0.000**	**0.010**	**0.004**
Hip RoM	32.3 (8.6)	25.6 (5.7)	42.1 (4.8)	37.5 (5.4)	**0.001**	**0.000**	0.240
Knee extension	6.0 (6.0)	13.1 (5.7)	4.0 (5.1)	8.3 (5.2)	**0.000**	**0.000**	0.104
Knee flexion	15.2 (4.2)	18.5 (6.1)	18.5 (4.8)	19.9 (5.0)	0.075	**0.005**	0.200
Knee RoM	9.2 (5.2)	5.4 (3.3)	14.6 (4.6)	11.6 (5.0)	**0.006**	**0.000**	0.523
Pelvic tilt	6.1 (5.4)	18.5 (4.3)	5.4 (5.5)	12.4 (5.1)	**0.000**	**0.000**	**0.000**
Pelvic obliquity up	1.8 (2.0)	2.5 (2.4)	3.2 (2.5)	3.1 (1.8)	0.575	**0.008**	0.300
mean FPA	-9.4 (7.3)	-10.0 (7.1)	-8.8 (5.1)	-9.1 (6.0)	0.786	0.372	0.887
Thorax tilt	10.2 (5.8)	8.2 (5.0)	8.2 (4.6)	6.3 (4.8)	0.165	**0.000**	0.941
Thorax lean	-3.0 (2.4)	-3.7 (2.7)	-3.1 (2.4)	-2.9 (2.8)	0.722	0.228	0.104
**Joint moments ($Nmkg^{-1}$)**							
Knee adduction_1	0.37 (0.13)	0.39 (0.15)	0.38 (0.11)	0.44 (0.17)	0.246	0.112	0.283
Knee adduction_2	0.30 (0.11)	0.31 (0.12)	0.34 (0.11)	0.35 (0.13)	0.778	**0.025**	0.936
Hip adduction_1	0.87 (0.22)	0.77 (0.19)	0.83 (0.14)	0.87 (0.22)	0.470	0.369	**0.047**
Hip adduction_2	0.83 (0.20)	0.71 (0.20)	0.82 (0.12)	0.80 (0.23)	0.156	0.201	0.088

Kinematic parameters (except FPA) and joint moments are the peak values during the stance phase of gait (standard deviation) averaged over the included trials. Negative values indicate extension, posterior tilt, external foot progression angle and ipsilateral thorax lean. Significant differences are bold printed.

Abbreviations: Int: interaction Group x Time; C1: cluster 1; C2: cluster 2; RoM: range of motion; FPA: foot progression angle; _1: during the first half of stance; _2: during the second half of stance.

Table S2: Preoperative and postoperative spatio-temporal, kinematic and kinetic outcome parameters and the p-values for the univariate analysis of variance (ANOVA) with normalized speed, age and BMI as covariates for comparison of the clusters to the healthy controls.

						**ANOVA**									
**Parameter**	**Pre**		**Post**		**HC**	**Pre**					**Post**				
	**C1**	**C2**	**C1**	**C2**		**Speed**	**Age**	**BMI**	**C1,HC**	**C2,HC**	**Speed**	**Age**	**BMI**	**C1,HC**	**C2,HC**
**Spatiotemporal**															
Walking speed (ms^{-1})	1.10 (0.15)	1.03 (0.19)	1.21 (0.11)	1.17 (0.17)	1.30 (0.14)		0.082	**0.010**	**0.000**	**0.000**		**0.007**	0.231	**0.043**	**0.005**
Step length (cm)	62.0 (4.3)	58.8 (8.5)	65.5 (5.2)	63.6 (7.4)	66.3 (5.6)	**0.000**	**0.001**	0.215	1.000	1.000	**0.000**	**0.000**	0.626	1.000	1.000
Cadence (steps per min)	108.1 (9.6)	106.3 (11.0)	111.6 (8.1)	110.6 (9.0)	116.7 (8.2)	**0.000**	**0.004**	0.380	1.000	0.433	**0.000**	**0.000**	0.892	1.000	1.000
Step width (cm)	9.1 (2.6)	10.7 (3.1)	8.0 (2.6)	9.4 (2.9)	8.7 (2.4)	0.237	0.720	**0.006**	1.000	1.000	0.990	0.395	**0.001**	0.193	1.000
**Kinematics (°)**															
Hip extension	-9.7 (8.9)	8.7 (6.6)	-14.5 (6.3)	-3.5 (7.9)	-14.4 (6.8)	0.072	0.399	**0.013**	1.000	**0.000**	0.335	0.231	**0.038**	1.000	**0.001**
Hip flexion	22.6 (5.0)	34.3 (4.6)	27.6 (7.2)	34.0 (4.9)	30.6 (6.4)	**0.008**	0.888	**0.017**	**0.001**	**0.021**	**0.019**	0.205	**0.002**	0.122	0.540
Hip RoM	32.3 (8.6)	25.6 (5.7)	42.1 (4.8)	37.5 (5.4)	45.0 (4.9)	**0.000**	0.347	0.452	**0.000**	**0.000**	**0.000**	0.835	0.399	0.679	**0.000**
Knee extension	6.0 (6.0)	13.1 (5.7)	4.0 (5.1)	8.3 (5.2)	2.4 (3.7)	0.721	0.249	0.295	0.231	**0.000**	0.055	0.338	0.346	1.000	**0.006**
Knee flexion	15.2 (4.2)	18.5 (6.1)	18.5 (4.8)	19.9 (5.0)	20.7 (4.6)	**0.000**	0.351	0.359	0.104	1.000	**0.028**	0.230	0.246	0.385	1.000
Knee RoM	9.2 (5.2)	5.4 (3.3)	14.6 (4.6)	11.6 (5.0)	18.3 (5.3)	**0.000**	0.725	0.809	**0.000**	**0.000**	**0.000**	0.784	0.802	0.197	**0.002**
Pelvic tilt	6.1 (5.4)	18.5 (4.3)	5.4 (5.5)	12.4 (5.1)	6.6 (5.4)	0.700	0.687	**0.007**	1.000	**0.000**	0.065	0.273	**0.013**	0.861	**0.005**
Pelvic obliquity up	1.8 (2.0)	2.5 (2.4)	3.2 (2.5)	3.1 (1.8)	4.0 (2.2)	0.061	0.337	0.540	**0.042**	0.956	**0.005**	0.369	0.261	1.000	1.000
mean FPA	-9.4 (7.3)	-10.0 (7.1)	-8.8 (5.1)	-9.1 (6.0)	-7.6 (4.5)	0.976	**0.019**	0.065	0.917	1.000	0.606	**0.008**	0.378	0.938	1.000
Thorax tilt	10.2 (5.8)	8.2 (5.0)	8.2 (4.6)	6.3 (4.8)	3.7 (4.3)	0.938	0.531	0.695	**0.000**	**0.016**	0.882	0.387	0.237	**0.001**	**0.041**
Thorax lean	-3.0 (2.4)	-3.7 (2.7)	-3.1 (2.4)	-2.9 (2.8)	-1.5 (1.9)	0.165	0.693	0.786	**0.015**	**0.001**	0.538	0.924	0.820	**0.031**	0.078
**Joint moments ($Nmkg^{-1}$)**															
Knee adduction_1	0.37 (0.13)	0.39 (0.15)	0.38 (0.11)	0.44 (0.17)	0.50 (0.15)	**0.039**	0.265	0.503	0.067	0.349	**0.000**	0.792	0.860	0.091	1.000
Knee adduction_2	0.30 (0.11)	0.31 (0.12)	0.34 (0.11)	0.35 (0.13)	0.43 (0.12)	0.474	0.065	0.562	**0.002**	**0.005**	0.097	0.076	0.561	**0.023**	0.077
Hip adduction_1	0.87 (0.22)	0.77 (0.19)	0.83 (0.14)	0.87 (0.22)	0.99 (0.16)	**0.007**	0.066	**0.004**	0.731	**0.004**	**0.000**	**0.001**	**0.006**	**0.047**	0.201
Hip adduction_2	0.83 (0.20)	0.71 (0.20)	0.82 (0.12)	0.80 (0.23)	0.90 (0.24)	0.602	0.225	**0.047**	0.997	**0.008**	0.474	0.096	**0.005**	0.424	0.074

Kinematic parameters (except FPA) and joint moments are the peak values during the stance phase of gait (standard deviation) averaged over the included trials. Negative values indicate extension, posterior tilt, external foot progression angle and ipsilateral thorax lean. Significant differences are bold printed.

Abbreviations: C1: cluster 1; C2: cluster 2; HC: healthy controls; BMI: Body Mass Index; RoM: range of motion; FPA: foot progression angle; _1: during the first half of stance; _2: during the second half of stance.
